# Observing resuscitative practice. A novice researcher’s experience of obtaining ethics approval

**DOI:** 10.1177/09697330231166071

**Published:** 2023-06-06

**Authors:** Katherine Riley, Luke Molloy, Val Wilson, Rebekkah Middleton

**Affiliations:** 8691 University of Wollongong, School of Nursing, Wollongong, NSW, Australia; 8691 University of Wollongong, School of Nursing, Wollongong, NSW, Australia; 8691 Prince of Wales Hospital, South Eastern Sydney Local Health District and South Western Sydney Nursing & Midwifery Research Alliance, Ingham Institute; 8691 University of Wollongong, School of Nursing, Wollongong, NSW, Australia

**Keywords:** ethnography, ethics committee, novice researcher, vulnerable groups, resuscitation, rural research, nurses

## Abstract

Undertaking research involving vulnerable groups, such as those requiring resuscitation involves careful analysis during the ethical review process. When a person lacks the capacity to make an informed choice about their participation in a research study, a waiver of consent offers an alternative. This paper is based on a doctoral research study using ethnography to explore the resuscitative practices and experiences of rural nurses through observation and interviews. This paper aims to explore the ethical issues raised by the Human Research Ethics Committee relating to consent of vulnerable patients requiring resuscitation within a rural context. In particular, the challenges of addressing risk (privacy) vs benefit (public) associated with a waiver of consent. This paper will consider why the rural context should be championed during the ethical review process, when decisions about public benefit are being deliberated. Utilising a communitarianism approach that advocates for greater rural representation during the ethical review processes will ensure that rural research involving vulnerable groups can be addressed safely and benefit not only the experiences and practices of rural nurses but also the wider rural communities they serve.

## Introduction

Human Research Ethics Committee’s (HREC) play a vital role in ensuring that human research studies are ethically acceptable and benefit the community by ensuring that aspects such as informed consent, privacy, minimising harm and confidentiality are clearly outlined in the research proposal.^1,2^ HREC’s are comprised of a broad representation of researchers, community members and members with specific expertise in ethics and research disciplines,^3^ with the aim of providing diverse and varied perspectives to the review process. When this is achieved, committees contribute positively to the human research ecosystem.^4^ In Australia, there are over 200 registered HRECs placed within universities, hospitals and smaller organisations across all states and territories. The majority of these committees are positioned in cities and urban locations, with a very small number located in regional centres.^5^ Their role is to ensure that research proposals involving human participants meet the ethical guidelines as set out in the National Statement on Ethical Conduct in Human Research.^6^

Communicating your research to others can be fraught with many challenges for a novice researcher. With little hands-on experience the researcher must become accustomed to how their methodology/methods inform the research design, whilst anticipating the common ethical issues intrinsic to the environment and people being researched.^3^ Whilst the ethical review process is often seen as an exciting milestone of a research study, for some, it can generate new challenges that place additional strain on the researcher, impose restrictions on the research design and lengthen the research timeline.^7^ Some of these challenges are associated with ethical issues inherent to the environment or the vulnerability of the participants within the study.^8,9^ Obtaining ethics approval for emergency research with a specific focus on the deteriorating patient and resuscitation have posed many unique ethical challenges for researchers and HREC’s.^10,11^ In particular, addressing consent issues within a research design when a participant lacks the capacity to provide informed consent.^12^

The ethnographic research study that will be addressed in this paper, aimed to explore the resuscitative experiences and practices of rural nurses. The study involved immersion in fieldwork at rural hospitals to document nurses’ behaviours and interactions up close, using observation and interviews. Prior to commencing the fieldwork, the study required ethics approval. This process took over 10 months to complete, due to concerns raised by the HREC regarding the risks associated with patient privacy versus benefits of the study.

This is an account of a novice researcher’s experience of obtaining ethics approval for their doctoral study. This paper describes the misunderstandings that transpired between the HREC and the researcher in a study positioned in an ethically high-risk clinical environment within a rural setting. This paper will explore the ethical issues raised by the HREC relating to consent of vulnerable patients requiring resuscitation. It presents a way of moving forward that may strengthen a researcher’s voice when navigating the ethics approval process involving observation of vulnerable populations. Whilst advocating that researchers and ethics committees consider the benefits of a communitarianism approach when designing and reviewing research protocol’s requiring a waiver of consent.

## The Australian rural context

Describing the rurality of a rural community can be difficult, as a range of factors, varying across a wide continuum, influences its composition. However, it generally encompasses smaller populations, greater distance and isolation from major cities with a lack of access to the full range of services and infrastructure.^13,14^ In Australia, 28% of the population live in rural and remote areas that service many diverse communities.^15^ Rural communities have statistically poorer health outcomes than urban areas, associated with higher rates of chronic disease, premature death and poorer access to healthcare.^16^ Therefore, there is pressing need for development of models of care to address rural health disparities and service the unique health needs of these communities.^17^

Rural hospitals support their communities within the constraints of geographical isolation and lack of direct access to specialist medical services. Despite these challenges, rural hospitals’ have a responsibility to ensure they provide a service that meets the workforce requirements of their communities.^18^ Rural hospitals are allocated emergency service delineation that requires nursing staff to be on-site 24 h with basic life support capability for adults and children, with some nursing staff trained in advanced life support. Whilst medical support is largely provided by local general practitioners as an on-call basis or via telehealth.^19^ The difficulties that rural nurses face is the expectation that stabilisation and treatment of critically ill patients occurs in a timely and responsive fashion as outlined in The National Safety and Quality Health Service Standards.^20^ Therefore, in the absence of onsite medical support, nurses are required to initiate lifesaving care for their deteriorating patient.

Recognition of, and response to, acute deterioration requires access to appropriately qualified, skilled and experienced clinicians.^20^ Rural nurses, compared to their urban colleagues, must possess a diverse set of skills to undertake autonomous and advanced clinical roles; as well as possess professional attributes of resilience, adaptability, creativity and innovation, in an environment of scarce resources and minimal support structures.^21^ In order to improve health disparities, we must first understand how rural nurses’ experience and practice these more complex clinical/professional skills needed for resuscitation in an environment with minimal medical support.^22^ This study aimed to address this specific need, and highlight the barriers and enablers rural nurses encounter when providing resuscitative care.

## The Research Case

This was an ethnographic study that used observation and interviews as the data collection methods. Ethnography is a descriptive and interpretative study of culture.^23,24^ The underlying assumption of the ethnographer is that social groups evolve into a culture that influences the ‘members’ view of the world and the way they construct their experiences.^25^ Ethnography is an effective methodology used in many health care settings to improve the understanding of cultures and sub-cultures that exist within these environments.^26^ The approach was chosen as a means to explore both the emic perspective – how rural nurses see their world and the etic perspective – how the researcher interprets the experience and practices of the culture,^25^ providing a rich understanding of how values and beliefs are embodied in rural nurses resuscitative practices and behaviours. Observing practice is an essential method in the ethnographic process that allows the researcher to gain a deeper understanding of the daily routines, rituals, patterns and trends in behaviours and practices of the ‘social group’ that is not always accessible with other methods.^23^

**Figure 1.**
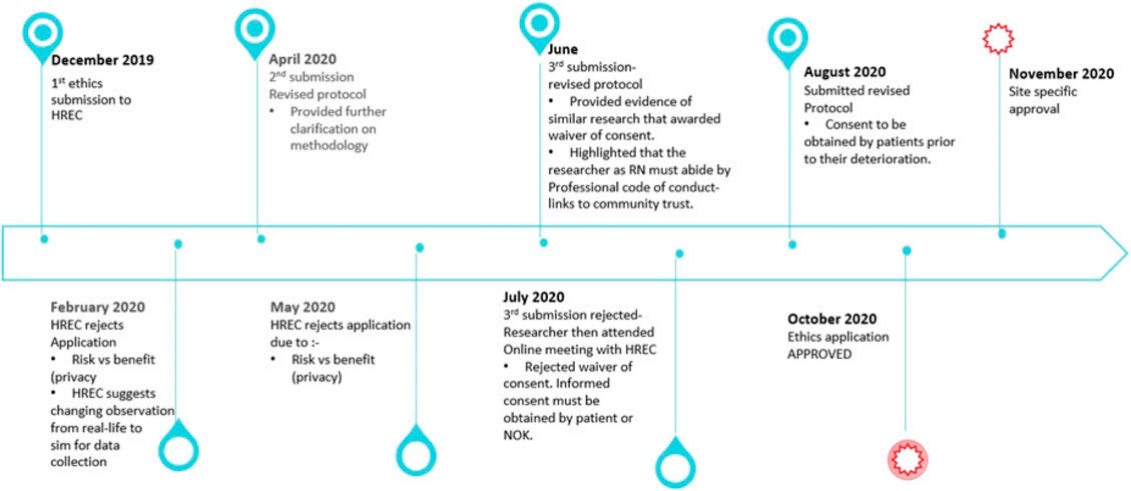
Ethics review timeline.

During a lengthy 10 month HREC review process (see Figure 1), the central concern raised by the HREC was the observation of nursing practice during a resuscitation. In the early stages of the review process, the researcher was able to facilitate solutions to the committee’s ethical concerns regarding the nurse as participant. However, the most protracted and challenging aspect of the review process was the ethical issues associated with patient privacy. The committee were concerned that observing nurses providing resuscitative care broadened the observational gaze of the researcher to include the patient being resuscitated. Although, the patients were not participants and no data would be collected about them, there were still ethical issues that would need to be resolved. The ethics committee were concerned that a patient would be at risk of potential harm or discomfort associated with being observed by an outsider (researcher), thereby breaching the patient’s right to privacy.

In healthcare research, obtaining informed consent from the patient ensures that a person’s right to make autonomous decisions is respected.^27^ However, patients in a position needing resuscitative care lack the capacity to consent due to the nature of their presentation, such as loss of consciousness or hypoxia, often in these cases, a waiver of consent can be approved by a HREC. In order to be granted a waiver of consent, the researcher must be able to demonstrate how they meet the requirements as outlined in Table 1.^1^ In July 2020, after two phases of rejections and a virtual meeting between the researcher and ethics committee, the HREC affirmed that a ‘*waiver of consent to observe patients without their consent [was] not approved’* as they were not satisfied that *‘the potential public benefit of the research substantially outweighs the risks to privacy’.*^28^ As a result, the HREC would only approve the study if written consent from the patient or their next of kin were obtained before clinical deterioration.

**Table 1.** Waiver of consent guideline^1^ (p.19).

If a waiver of consent is to be approved, a HREC must be satisfied that
• involvement in the research carries no more than low risk
• the benefits from the research justify any risks of harm associated with not
seeking consent
• it is impracticable to obtain consent
• there is no known or likely reason for thinking that participants would not have
consented if they had been asked
• there is sufficient protection of their privacy

### Epistemological and methodological issues

There are many published papers that describe the challenges that researchers experience in obtaining ethics approval for ethnographic studies.^9,29–32^ Many ethnographic researchers undertaking studies in healthcare settings have identified considerable hurdles where issues of vulnerability such as a person’s cognitive impairment,^8^ ill health^33^ or young age^34^ are involved. Some of the reported challenges highlight an underlying disconnect between HREC’s and researchers’ epistemological viewpoints.^9,31,35^ Bell & Wynn^29^ helps to unpack this notion by asserting that an ethnographer’s epistemological stance aligns within the social science realm and adopts a subjective and interpretivist position, whilst HREC’s reside within a biomedical paradigm that promotes the values of ‘objectivity’ and a positivist orientation. It has been suggested that the disconnect between these opposing views can create an ‘us and them’ dynamic perpetuated by feelings of being misunderstood by the members of the HREC,^7^ potentially influencing the ‘direction of ethnographic inquiry, the knowledge produced and the safety of both participants and researchers’.^36^ (p729)

The following quote from the committee highlights the possible epistemological and/or methodological disconnect between the researcher and the HREC. *‘Is it possible to obtain the necessary data by observing simulated resuscitation scenarios and/or by interviewing nurses following a resuscitation event?’*^28^ In addressing this concern, the researcher interpreted this line of questioning to mean that the committee were unclear of the methodology being used; since observing a simulated resuscitation would not replicate a real-life resuscitation event or the culture within it. In doing so, the researcher provided further clarification about ethnography and why this methodology met the needs of the study aims.

### Informed consent and Vulnerable populations

The practice of informed consent is a dynamic process that requires the application of basic principles of self-determination, competence, and voluntariness.^37^ Informed consent is fundamental to the ethical principles of respect for persons and autonomy and is a critical requirement when undertaking research. However, it can be a highly problematic process in a variety of research settings, particularly in vulnerable populations such as the critically unwell.^11,33,38^ Due to a potential lack of capacity to make informed decisions and the risk for coercion, vulnerable participants' involvements in studies are thoroughly examined during the ethical review process.^12^ In theory, to ensure that patients are protected from the potential risks of their involvement, informed consent should be obtained. However, challenges occur when a person’s capacity to consent is affected. For consent to be informed, requires competency and capacity to understand the information provided by the researcher and the implications of participation, so an informed decision on their involvement can be made.^1^ People that present to emergency departments that are at risk of requiring resuscitation, temporarily lack the decision-making capacity to give informed consent due to symptoms such as pain, hypoxia, loss of consciousness or the effects of alcohol/elicit substances.^10^ In addition, time constraints associated with the nature of the condition and the urgent need for stabilising treatment compromises a patient’s ability to carefully way up the risks and benefits of their involvement.^11^ Obtaining consent from a patient’s legal guardian is an alternative that can be approved by an ethics committee; however, during a time-critical stressful situation this is impractical, and can be perceived as intrusive and insensitive for family.^10^ It is for these reasons that alternate methods should be considered such as a waiver of consent. In Australia, a waiver of consent can be granted in clinical research under specific circumstances as outlined in Table 1. In doing so, HREC’s must weigh up the patients right to consent and the societal benefits of the study.^33^

When a waiver of consent is approved by an ethics committee it affords a vulnerable persons right to participate in research, whilst fulfilling the ethical principle of justice.^33^ It also ensures that non-maleficence is upheld by safeguarding a person’s inability to make an informed decision regarding consent.^36^ In this proposed study, a waiver of consent was denied by the HREC – stating that the ‘*public benefits of the research [did not] substantially outweigh the risks to privacy’.*^28^ Instead of approving a waiver of consent, the ethics committee would only approve informed consent of a patient before the patient needed resuscitation.

In addressing the waiver of consent requirements (see Table 1), the area most challenging to tackle was the risk (privacy) vs benefit. During the study design process, the researcher assumed rural communities would support the study because it had the potential to benefit them and their hospitals service delivery during resuscitations. However, without prior rural community involvement in the study design process, it was impossible to provide evidence to the HREC that the community would have considered this research as having ‘public benefit’.

## Discussion

It is critical that research proposals are scrutinised by ethics committees, to safeguard vulnerable participants such as the critically unwell from potential harm.^39^ When research has social value, involves minimal risk and it is otherwise impractical to consent due to issues with capacity and time constraints, then a waiver of consent offers an alternative.^36^ In this study, the HREC denied a waiver of consent based on concerns that the risk to privacy from being observed by the researcher far outweighed the public benefits of the observational data collected of rural nurses’ resuscitative practices. This resulted in rigid and impractical modifications to the consent process of patients; and instead of providing protection; it increased the potential for risk of harm or excluded them entirely from the research study. O’Connor^8^ asserts that HREC should consider how their decisions such as denying a waiver of consent influences other ethical principles, such as non-maleficence and justice. This was pertinent in this study, whereby the HREC requested consent be obtained during a narrow therapeutic window between a patient presenting acutely unwell to ED but before clinical deterioration requiring resuscitation. Attempting to consent patients during these critical moments has the potential of causing unnecessary harm and negates the ethical principle of non-maleficence.^32^ Although, critically ill people are considered a vulnerable group, under the ethical principle of justice – they have the right to benefit from medical research that has the potential to improve practice and patient outcomes.^33^

Whilst epistemological and methodological indifferences could have been the catalyst for some of the misunderstandings between researcher and the HREC, a lack of understanding of the rural health context may offer a new explanation. When rural communities are included in the catchment of urban HREC’s as seen in this study, there is a risk that committee decisions will be influenced by an urban centric lens.^40,41^ This arises when ethics committees approach rural research and associated communities with preconceived views and assumptions based on their urban experiences and standpoints; rather than acknowledging that rural communities have distinct cultures that are influenced by how people live and work.^13,42^ Simpson^41^ summarises these sentiments by acknowledging that in order to determine whether ethical approaches frequently used remain relevant or operate in the same way in a context that may be somewhat different, such as rural settings, we need to critically challenge the underlying assumptions that support them. In addition, ethics committees that are based in urban communities, may have limited understanding of the diversity and complexity of the rural context when weighing up the risk versus benefits of rural research. As a result, one cannot assume that the frameworks, theories and practices utilised by ethics committees are necessarily translatable to rural communities and the rural health context.^41^ Raising the question, are urban HRECs in the best position to assert what is beneficial for a rural community?

### Moving forward

To address these issues, ethics and ethical research needs to be reframed, away from a formal proceduralism approach and towards an ideal that takes into account the health disparities present in rural communities; whilst respecting the subtleties that local values and ethos play in influencing rural communities.^41^ Researchers and ethics committees have much to do in advocating for the health needs of rural communities. Nandra^12^ highlights that research involving acutely and/or critically unwell patients can be achieved with a waiver of consent if the researcher engages community and continuously audits practice. When defining the issues affecting their members, communities are frequently their own best spokespersons, so it makes sense that when researchers involve communities, the relevance of the research can be improved^43^

Rural communities voices should be championed when decisions about rural for rural are made by having rural representation at the table either literally or symbolically. Researchers and ethics committees can achieve this by adopting a communitarianism stance. This approach promotes the importance of community consultation and involvement, whilst bringing about the benefits of power sharing.^41,44^ Holzer^43^ study highlights the importance of this relationship by recognising that community engagement symbolises respect for a communities values and interest, and may provide a twofold effect by serving to assuage participant risk by informing researchers and HREC’s the contextual meanings of risk and benefit associated with a communities sentiments 45,46; whilst being responsive to the cultural landscape of rural health services^47^

Whilst there is literature to support the importance of community engagement when designing research studies involving vulnerable groups.^45,46^ It remains unclear what constitutes meaningful involvement with community and how these can be evaluated.^48,49^ Despite these challenges, there are many published examples of community engagement initiatives addressing a wide cohort of participant groups such as research involving people with dementia,^45^ palliative care^50^ and substance use disorder.^51^ Notably to date, community engagement initiatives associated with rural communities and rural research have received scant attention in the research literature.

This paper explored the ethical challenges the researcher experienced during the ethical review process of an ethnographic study, exploring the resuscitative experiences and practices of rural nurses. Although this studies context lies within a specific healthcare area, the paper addresses issues that could be encountered by other researchers when advocating for a waiver of consent; such considerations could be utilised in other ethics applications particularly when under-represented and/or vulnerable groups are involved.

## Conclusion

Barriers to undertaking non-experimental resuscitative research such as the ethical challenges experienced by the researcher obtaining ethics approval, have the capacity to impact research being undertaken with vulnerable populations that are unable to consent, whilst diminishing the advancement of research in rural settings. Researchers have a role in upskilling themselves and ethics committees when advocating for vulnerable populations during an ethics review process. Addressing these issues will mean that future rural research questions can be addressed safely and benefit not only the experiences and practices of rural nurses but also the wider rural communities they serve.
